# An Improved Teaching-Learning-Based Optimization with the Social Character of PSO for Global Optimization

**DOI:** 10.1155/2016/4561507

**Published:** 2016-01-14

**Authors:** Feng Zou, Debao Chen, Jiangtao Wang

**Affiliations:** School of Physics and Electronic Information, Huaibei Normal University, Huaibei 235000, China

## Abstract

An improved teaching-learning-based optimization with combining of the social character of PSO (TLBO-PSO), which is considering the teacher's behavior influence on the students and the mean grade of the class, is proposed in the paper to find the global solutions of function optimization problems. In this method, the teacher phase of TLBO is modified; the new position of the individual is determined by the old position, the mean position, and the best position of current generation. The method overcomes disadvantage that the evolution of the original TLBO might stop when the mean position of students equals the position of the teacher. To decrease the computation cost of the algorithm, the process of removing the duplicate individual in original TLBO is not adopted in the improved algorithm. Moreover, the probability of local convergence of the improved method is decreased by the mutation operator. The effectiveness of the proposed method is tested on some benchmark functions, and the results are competitive with respect to some other methods.

## 1. Introduction

Global optimization is a concerned research area in science and engineering. Many real-world optimization applications can be formulated as global optimization problems. To efficiently solve the global optimization problems, the efficient and robust optimization algorithms are needed. The traditional methods often fail to solve complex global optimization problems. A detailed overview of the research progress in deterministic global optimization can be found in [1]. To overcome the difficulties of traditional methods, some well-known metaheuristics are developed for solving global optimization problems during the last four decades. Among existing metaheuristics, particle swarm optimization (PSO) algorithm [2] plays very important role in solving global optimization problems. PSO inspired by the social behaviors of birds has been successfully utilized to optimize continuous nonlinear functions [3], but the standard PSO algorithm (SPSO) might trap into local optima when solving complex multimodal problems. To improve the global performance of PSO, some variants are developed. Linear decreasing inertia weight particle swarm optimization (LDWPSO) [4] was introduced by Shi and Eberhart to overcome the lack of velocity control in standard PSO; the inertia weight of the algorithm decreases from a large value to a small one with increasing of evolution. To improve the convergence accuracy of PSO, some modified operators are adopted to help the swarm escape from the local optima especially when the best fitness of the swarm is not changed in some continuous iteration [5–8]. To improve the convergence speed of PSO, some modifications for updating rule of particles were presented in [9], and the improved algorithm was tested on some benchmark functions. Adaptive particle swarm optimization algorithm was introduced by Zhan to improve the performance of PSO, many operators were proposed to help the swarm jump out of the local optima and the algorithms have been evaluated on 12 benchmark functions [10, 11]. Some detail surveys of development to PSO are introduced for the interested readers in [12–14].

Though swarm intelligence optimization algorithms have been successfully used to solve global optimization problems, the main limitation of the previously mentioned algorithms is that many parameters (often more than two parameters) should be determined in updating process of individuals, and the efficiency of algorithms are usually affected by these parameters. For example, there are three parameters (*w*, *c*_1_, and *c*_2_) that should be determined in updating equations of PSO. Moreover, the optimal parameters of the algorithms are often difficult to be determined. To decrease the effects of parameters for the algorithms, teaching-learning-based (TLBO) algorithm [15] is proposed recently, and it has been used in some real applications [16–19]. Some variants of the TLBO algorithm have been presented for improving the performance of the original TLBO [20, 21].

Under the framework of population-based optimizations, many variations of evolutionary optimization algorithms have been designed. Each of these algorithms performs well in certain cases and none of them are dominating one another. The key reason for employing the hybridization is that the hybrid algorithm can take advantage of the strengths of each individual technique while simultaneously overcoming its main limitations. On top of this idea, many hybrid algorithms have been presented [22–27]. In the paper, to improve the performance of TLBO algorithm for solving global optimization problems, an improved TLBO algorithm with combining of the social character of PSO, named TLBO-PSO, is proposed. In the improved TLBO algorithm, the teacher improves not only the performance of the mean grade of the whole class but also the performance of every student. The proposed algorithm has been evaluated on some benchmark functions, and the results are compared with some other algorithms.

The paper is organized as follows.  Section 2 provides a brief description of the standard PSO algorithm. Original teaching-learning-based (TLBO) algorithm is introduced in Section 3.  Section 4 provides the detail procedure of the improved teaching-learning-based optimization algorithm with combining of the social character of PSO (TLBO-PSO). Some experiments are given in Section 5.  Section 6 concludes the paper.

## 2. Particle Swarm Optimizers

In the standard PSO (SPSO) algorithm, a swarm of particles are represented as potential solutions; each particle searches for an optimal solution to the objective function in the search space. The *i*th particle is associated with two vectors, that is, the velocity vector *V*_*i*_ = [*v*_*i*_^1^, *v*_*i*_^2^,…, *v*_*i*_^*D*^] and the position vector *X*_*i*_ = [*x*_*i*_^1^, *x*_*i*_^2^,…, *x*_*i*_^*D*^], where *D* is the dimensions of the variable. Each particle dynamically updates its position in a limited searching domain according to the best position of current iteration and the best position which it has achieved so far. The velocity and position of the *i*th particle are updated as follows:

(1) vid=wvid+c1r1Pbestid−xid+c2r2Gbestd−xid,

(2) xid=xid+vid,

where *c*_1_ and *c*_2_ are the acceleration coefficients, the value of them often equals 2, *r*_1_ and *r*_2_ are random numbers between 0 and 1. *w* is the inertia weight which influences the convergent character of the swarm. Large inertial weight benefits global searching performance, while small one facilitates local searching. *x*_*i*_^*d*^ is the position of the *i*th particle on the *d*th dimension, *Pbest*_*i*_^*d*^ is the best position which the *i*th has achieved so far, and *Gbest* ^*d*^ is the best position of the swarm in current iteration. In general, the velocity of all the particles is limited by the maximum velocity (*V*_max_), and positions of them are limited by the maximum position (*P*_max_) and the minimum position (*P*_min_). To improve the performance of PSO, the method LDWPSO in which the inertia weight decreases linearly from a relatively large value to a small one is proposed [4], and the changed weight with evolution iteration is shown as follows:

(3) $$\begin{array}{c} w=w_{max}-gen*\frac{w_{max}-w_{min}}{\max gen}, \end{array}$$

where *w*_max_ = 0.9, *w*_min_ = 0.4 are the maximum and minimum values of inertia weight, respectively. gen is the current generation, max⁡gen is the maximum evolutionary iteration. The adaptive weights make swarm have good global searching ability at the beginning of iterations and good local searching ability near the end of runs.  Algorithm 1 presents the detail steps of LDWPSO algorithm.

## 3. Teaching-Learning-Based Optimization (TLBO) Algorithm

TLBO is one of the recently proposed population-based algorithms and it simulates the teaching-learning process of the class [15]. The main idea of the algorithm is based on the influence of a teacher on the output of learners in a class and the interaction between the learners. The TLBO algorithm does not require any specific parameters. TLBO requires only common controlling parameters like population size and number of generations for its working. The use of teaching in the algorithm is to improve the average grades of the class. The algorithm contains two phases: teacher phase and learner phase. The detail description of the algorithm can be found in [15]. In this paper, only the two main phases of TLBO are introduced as follows.

### 3.1. Teacher Phase

In the teacher phase of TLBO algorithm, the task of the teacher is to increase the mean grades of the class. Suppose that an objective function is *f*(*X*) with *n*-dimensional variables, the *i*th student can be represented as *X*_*i*_ = [*x*_*i*1_, *x*_*i*2_,…, *x*_*in*_]. At any iteration *g*, assume that the population size of the class is *m*, the mean result of students in current iteration is *X*_*g*mean_ = (1/*m*)[∑_*i*=1_^*m*^ *x*_*i*1_, ∑_*i*=1_^*m*^ *x*_*i*2_,…, ∑_*i*=1_^*m*^ *x*_*in*_]. The student with the best fitness is chosen as the teacher of current iteration; it is represented as *X*_teacher_. All the students will update their position as follows:

(4) $$\begin{array}{c} X_{i,new}=X_{i,old}+r_{1}\left(\;X_{teacher}-T_{F}X_{gmean}\;\right), \end{array}$$

where *X*_*i*,new_ and *X*_*i*,old_ are the new and the old position of the *i*th student and *r*_1_ is the random number in the range [0,1]. If the new solution is better than the old one, the old position of individual will be replaced by the new position. The value of *T*_*F*_ is randomly decided by the algorithm according to

(5) $$\begin{array}{c} T_{F}=round\left[\;1+\mathrm{rand}\left(\;0,1\;\right)\left\{\;2-1\;\right\}\;\right]. \end{array}$$

### 3.2. Learner Phase

In learner phase of TLBO algorithm, learners can increase their knowledge from others. A learner interacts randomly with other learners for enhancing his or her knowledge. The learning of learner phase can be expressed as follows. For the *i*th individual *X*_*i*_ in the *j*th generation, randomly select the *k*th individual *X*_*k*_ which is different from *X*_*i*_, and the updated formula of *X*_*i*_ is defined in (6) and (7).

If *X*_*i*_ is better than *X*_*k*_ according to their fitness, then

(6) $$\begin{array}{c} X_{i,new}=X_{i,old}+r_{i}\left(\;X_{i}-X_{k}\;\right). \end{array}$$

Else

(7) $$\begin{array}{c} X_{i,new}=X_{i,old}+r_{i}\left(\;X_{k}-X_{i}\;\right), \end{array}$$

where *r*_*i*_ is the random number in the range [0,1].

If the new position *X*_*i*,new_ is better than the old one *X*_*i*,old_, the old position *X*_*i*,old_ is replaced by the new *X*_*i*,new_; otherwise, the position of the *i*th individual is not changed. The detail algorithm is shown in Algorithm 2.

## 4. Teaching-Learning-Based Optimization Algorithm with PSO (TLBO-PSO)

### 4.1. The Main Idea of TLBO-PSO

As previously reviewed, the main idea of TLBO is to imitate the teaching-learning process in a class. The teacher tries to disseminate knowledge among the learners to increase the knowledge level of the whole class, and the learners also study knowledge from the others to improve its grade. Algorithm displays that all individuals update their positions based on the distance between one or two times of the mean solution and the teacher in teacher phase. An individual also renewed its position based on the distance between it and a randomly selected individual from the class. Equation (4) indicates that the teacher only improves the grades of the students by using the mean grade of the class; the distance between the teacher and the students is not considered in teacher phase. In PSO, the difference between the current best individual and the individual can help the individual improve its performance. Based on this idea, the method in PSO is introduced into TLBO to improve the learning efficiency of the TLBO algorithm. The main change for TLBO is represented in updating equation. Equation (4) in TLBO is modified as

(8) Xi,new=Xi,old+r1Xteacher−TFXgmean+r2Xteacher−Xi,old,

where *r*_1_, *r*_2_ are the random number in the range [0, 1]. With this modification, the performance of TLBO algorithm might be improved.

In original TLBO algorithm, all genes of each individual should be compared with those of all other individuals for removing the duplicate individual. This operator is a heavy computation cost process, and the function evaluations required are not clearly known. In our opinion, duplication testing is not needed in every generation especially in the beginning of evolution. Assume that individuals *i* and *k* have the same genes in *t* generation, and the new position of these two individuals might be different when *T*_*F*_ is a random number (1 or 2). At the beginning of evolution, all individuals generate better positions easily. Random operator for *T*_*F*_ may benefit for maintaining diversity of class, but, in the anaphase of evolution, the positions of individuals might be close to each other. When the mean solution equals the best solution, the individual does not change (*T*_*F*_ = 1) or the large change of genes might destroy the individual in large degree (*T*_*F*_ = 2) so that the better individual is difficult to be generated. To decrease the computational effort of comparing all the individuals, the removing of the duplicate individual process in the original TLBO is deleted in the improved TLBO algorithm and a mutation operator according to the best fitness of successive generations is introduced in the paper. If the best fitness of continuous *n* generations is not changed or changed slightly, an individual will be randomly selected according to mutation possibility *p*_*c*_ to be mutated. To maintain the global performance of the algorithm, the best individual is not mutated. The subroutine for the mutation is described as shown in Algorithm 3.

In Algorithm 3, *n* is the setting generation, *p*_*c*_ is the mutation possibility, and *ε* is a small number which is given by the designer. *α* is a mutation parameter between 0.01 and 0.1.

### 4.2. The Steps of TLBO-PSO

**Step 1 .**

Set the maximum *X*_max_ and minimum *X*_min_ of position, the maximal evolution generation genmax, mutation possibility *p*_*c*_ and mutation parameter *α*, the population size popsize, and the dimension size of the task. Initialize the initial population pop as follows:

(9) $$\begin{array}{c} pop=X_{min}+r*\left(\;X_{max}-X_{min}\;\right), \end{array}$$

where *r* is the random number in the range [0,1].

**Step 2 .**

Evaluate the individual, select the best individual *X*_teacher_ as the teacher, and calculate the mean solution *X*_*g*mean_ of the population.

**Step 3 .**

For each individual, update its position according to (8). If *X*_*i*,new_ is better than *X*_*i*,old_, then *X*_*i*,old_ = *X*_*i*,new_.

**Step 4 .**

For each individual, randomly select another individual, update its position according to (6) and (7), and choose the better solution from *X*_*i*,old_ and *X*_*i*,new_ as the new position of the individual.

**Step 5 .**

Execute mutation operator for the population according to Algorithm 3.

**Step 6 .**

If the ended condition of TLBO-PSO is not satisfied, the algorithm will go back to Step 2, or it is terminated.

## 5. Simulation Experiments

To test the performance of the improved TLBO-PSO algorithm, nine benchmark functions are simulated in this section. These nine benchmark functions are listed in Table 1. In particular, in order to compare the performance of the proposed TLBO-PSO with variants of TLBO and PSO, CLPSO [22], TLBO [15], and ETLBO [21] are selected and simulated.

### 5.1. Parameters Setting

To reduce statistical errors, each function in the paper is independently simulated 30 runs, and their mean results are used in the comparison. The value of function is defined as the fitness function. All the experiments are carried out on the same machine with a Celeron 2.26 GHz CPU, 512-MB memory, and Windows XP operating system with Matlab 7.0. All functions are simulated in 10 and 30 dimensions. The nine functions are summarized in Table 1. “Range” is the lower and upper bounds of the variables. “*f*_min_” is the theory global minimum solution. “Acceptance” is the acceptable solutions of different functions. For CLPSO, TLBO, and ETLBO algorithms, the training parameters are the same as those used in the corresponding references except that the maximal FEs are 50000 and the size of population is 30. The size of elitism is 2 in ETLBO algorithm. When the best fitness is changed to be smaller than *ε*, the large value of mutation will possibly be chosen. In our experiments, the mutation possibility *p*_*c*_ is 0.6, *ε* is 0.01, and *α* is 0.05.

### 5.2. Comparisons on the Solution Accuracy

The performance of different algorithms for 10- and 30-dimensional functions in terms of the best solution, the mean (Mean), and standard deviation (STD) of the solutions obtained in the 30 independent runs is listed in Tables 2 and 3. Boldface in the tables indicates the best solution among those obtained by all four contenders.

The results for 10-dimensional functions in Table 2 display that the improved TLBO (TLBO-PSO) outperforms all the other algorithms in terms of the mean best solutions and standard deviations (STD) for functions *f*_1_, *f*_2_, *f*_3_, and *f*_4_. For function *f*_5_, ETLBO has the best performance. For function *f*_6_, the mean best solution of TLBO-PSO is the best, and the STD of TLBO is the smallest. For functions *f*_7_ and *f*_9_, CLPSO has the best performance. For function *f*_8_, three TLBOs have the same solutions in terms of the mean best solutions and standard deviations (STD).

The results for 30-dimensional functions in Table 3 indicate that TLBO-PSO also has the best performance in terms of the mean best solutions and standard deviations (STD) for functions *f*_1_, *f*_2_, *f*_3_, and *f*_4_ with 30 dimensions. TLBO has the best performance for function *f*_5_. For function *f*_6_, TLBO and ETLBO have the same performance in terms of the mean best solutions and standard deviations (STD). For function *f*_7_, the mean best solution of TLBO is the smallest, and the standard deviation of TLBO-PSO is the smallest. Three TLBOs have the same solutions for functions *f*_8_ and *f*_9_.

### 5.3. Comparisons on the Convergence Speed and Successful Ratios

The mean number of function evolutions (FEs) is often used to measure the convergence speed of algorithms. In this paper, the mean value of FEs is used to measure the speed of all algorithms. The average FEs (when the algorithm is globally convergent) of all algorithms for nine functions with 10 and 30 dimensions are shown in Tables 4 and 5. Boldface in the tables indicates the best result among those obtained by all algorithms. When the algorithm is not globally convergent in all 30 runs, the mFEs is represented as “NaN.” The successful ratios of different algorithms for the nine functions are also shown in the tables.

**Figure 1:**
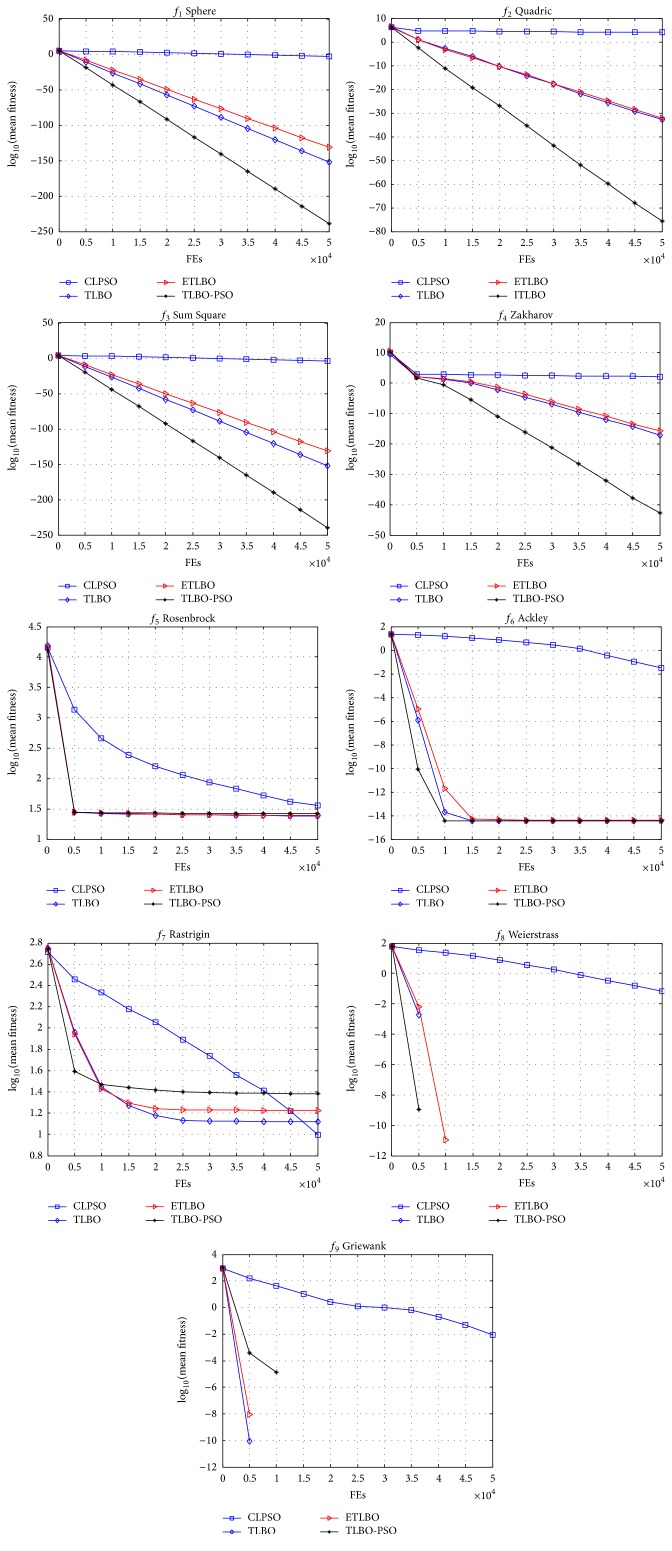
Convergence performance of the four different methods for 30-dimensional functions.

Tables 4 and 5 display that mFEs of TLBO-PSO are the smallest for large part of functions except that for function *f*_5_. The merit in terms of mean FEs for 10-dimensional function *f*_5_ with ETLBO is the best and the four algorithms cannot converge to the acceptable solution for 30-dimensional function. For function *f*_6_, all algorithms can converge to the global optima with 100% successful ratios on 30 dimensions except CLPSO. For function *f*_7_, CLPSO can converge to optima with 100% successful ratios for 10-dimensional function. They all cannot converge to 30-dimensional function except that the successful ratio of TLBO-PSO is 10%. The convergence speed of average best fitness of four algorithms is shown in Figure 1. The figures indicate that the TLBO-PSO has the best performance for large part of functions. According to the theorem of “no free lunch” [28], one algorithm cannot offer better performance than all the others on every aspect or on every kind of problem. This is also observed in our experimental results. For example, the merit of TLBO-PSO is worse than those of TLBO and ETLBO for 10-dimension function but it is better than large part of algorithms for other functions.

## 6. Conclusions 

An improved TLBO-PSO algorithm which is considering the difference between the solutions of the best individual and the individual that want to be renewed is designed in the paper. The mutation operator is introduced to improve the global convergence performance the algorithm. The performance of TLBO-PSO is improved for larger part of functions especially for 30-dimension functions in terms of convergence accuracy and mFEs. The local convergence of TLBO-PSO is major caused by the lost diversity in the later stage of the evolution.

Further works include researches into adaptive selection of parameters to make the algorithm more efficient. Moreover, it needs to seek a better method to improve the TLBO algorithm for functions with optimal parameters displaced from all zeroes. Furthermore, the algorithm may be applied to constrained, dynamic optimization domain. It is expected that TLBO-PSO will be used in real-world optimization problems.

## Figures and Tables

**Algorithm 1:**
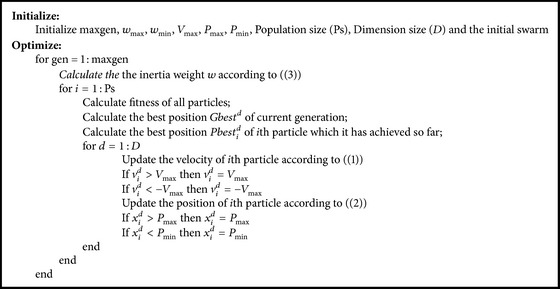
LDWPSO algorithm.

**Algorithm 2:**
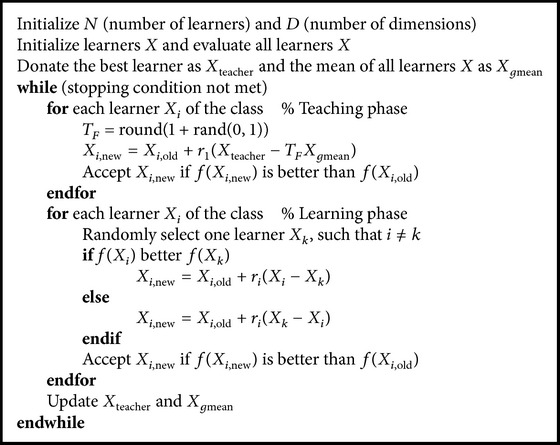
TLBO().

**Algorithm 3:**
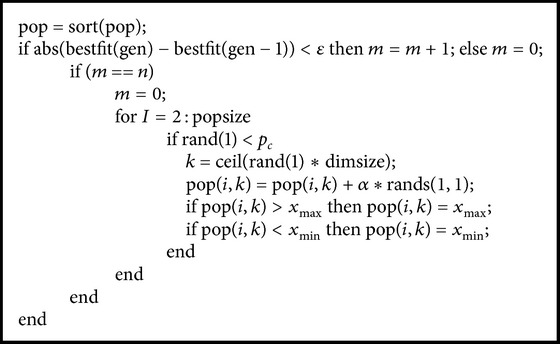
Subroutine for mutation.

**Table 1:** Nine tested functions.

Function	Formula	Range	*f*_min_	Acceptance
*f*_1_ (Sphere)	*f*_1_(*x*) = ∑_*i*=1_^*D*^ *x*_*i*_^2^	[−100,100]	0	1*E* − 6

*f*_2_ (Quadric)	*f*_2_(*x*) = ∑_*i*=1_^*D*^(∑_*j*=1_^*i*^ *x*_*j*_)^2^	[−100,100]	0	1*E* − 6

*f*_3_ (Sum Square)	*f*_3_(*x*) = ∑_*i*=1_^*D*^ *ix*_*i*_^2^	[−100,100]	0	1*E* − 6

*f*_4_ (Zakharov)	$f_{4}\left(x\right)=\sum_{i=1}^{D}x_{i}^{2}+{\left(\sum_{i=1}^{D}0.5ix_{i}\right)}^{2}+{\left(\overset{D}{\underset{i=1}{\sum }}0.5ix_{i}\right)}^{4}$	[−10,10]	0	1*E* − 6

*f*_5_ (Rosenbrock)	*f*_5_(*x*) = ∑_*i*=1_^*D*−1^[100(*x*_*i*_^2^ − *x*_*i*+1_)^2^ + (*x*_*i*_ − 1)^2^]	[−2.048,2.048]	0	1*E* − 6

*f*_6_ (Ackley)	$f_{6}\left(x\right)=20-20\exp\left(-\frac{1}{5}\sqrt{\frac{1}{D}\sum_{i=1}^{D}x_{i}^{2}}\right)-\exp\left(\frac{1}{D}\sum_{i=1}^{D}\cos\left(2\pi x_{i}\right)\right)+e$	[−32.768,32.768]	0	1*E* − 6

*f*_7_ (Rastrigin)	*f*_7_(*x*) = ∑_*i*=1_^*D*^(*x*_*i*_^2^ − 10cos⁡(2*πx*_*i*_) + 10)	[−5.12,5.12]	0	1*E* − 6

*f*_8_ (Weierstrass)	$\begin{array}{c} f_{8}\left(x\right)=\sum_{i=1}^{D}\left(\sum_{k=0}^{k_{max}}\left[a^{k}\cos\left(2\pi b^{k}\left(x_{i}+0.5\right)\right)\right]\right)-D\sum_{k=0}^{k_{max}}\left[a^{k}\cos\left(2\pi b^{k}\times 0.5\right)\right]\\ \begin{array}{ccc} a=0.5 & b=3 & k_{max}=20 \end{array} \end{array}$	[−0.5,0.5]	0	1*E* − 1

*f*_9_ (Griewank)	$f_{9}\left(x\right)=\sum_{i=1}^{D}\frac{x_{i}^{2}}{4000}-\prod_{i=1}^{n}\cos\left(\frac{x_{i}}{\sqrt{i}}\right)+1$	[−600,600]	0	1*E* − 1

**Table 2:** The mean best solutions and standard deviations by various methods for 10D functions.

Methods	CLPSO		TLBO		ETLBO		TLBO-PSO	
	Mean	STD	Mean	STD	Mean	STD	Mean	STD
*f*_1_	4.68*E* − 19	4.90*E* − 19	2.12*E* − 185	0.00*E* + 00	1.51*E* − 167	0.00*E* + 00	1.07**E** − 314	0.00**E** + 00
*f*_2_	6.73*E* − 01	4.40*E* − 01	5.79*E* − 80	1.71*E* − 79	5.10*E* − 78	1.22*E* − 77	9.62**E** − 172	0.00**E** + 00
*f*_3_	1.14*E* − 20	8.02*E* − 21	1.02*E* − 185	0.00*E* + 00	5.91*E* − 169	0.00*E* + 00	2.65**E** − 315	0.00**E** + 00
*f*_4_	3.32*E* − 03	3.25*E* − 03	4.44*E* − 87	1.36*E* − 86	9.99*E* − 85	1.89*E* − 84	5.04**E** − 182	0.00**E** + 00
*f*_5_	2.25*E* + 00	1.00*E* + 00	6.54*E* − 01	6.56*E* − 01	2.76**E** − 01	2.78**E** − 01	4.18*E* + 00	7.46*E* − 01
*f*_6_	3.75*E* − 10	2.83*E* − 10	3.55*E* − 15	0.00**E** + 00	3.20*E* − 15	1.12*E* − 15	2.84**E** − 15	1.50*E* − 15
*f*_7_	1.63**E** − 09	2.42**E** − 09	2.17*E* + 00	1.86*E* + 00	2.33*E* + 00	1.76*E* + 00	5.26*E* + 00	4.63*E* + 00
*f*_8_	1.16*E* − 11	1.55*E* − 11	0.00**E** + 00	0.00**E** + 00	0.00**E** + 00	0.00**E** + 00	0.00**E** + 00	0.00**E** + 00
*f*_9_	1.06**E** − 03	2.29**E** − 03	9.96*E* − 03	1.02*E* − 02	8.45*E* − 03	1.61*E* − 02	3.54*E* − 02	5.27*E* − 02

**Table 3:** The mean best solutions and standard deviations by various methods for 30D functions.

Methods	CLPSO		TLBO		ETLBO		TLBO-PSO	
	Mean	STD	Mean	STD	Mean	STD	Mean	STD
*f*_1_	1.50*E* − 03	5.04*E* − 04	1.63*E* − 152	2.38*E* − 152	5.70*E* − 132	1.13*E* − 131	6.67**E** − 240	0.00**E** + 00
*f*_2_	9.37*E* + 03	2.23*E* + 03	1.78*E* − 33	3.34*E* − 33	4.58*E* − 33	5.40*E* − 33	1.98**E** − 76	4.73**E** − 76
*f*_3_	1.39*E* − 04	4.19*E* − 05	1.45*E* − 152	2.79*E* − 152	7.13*E* − 132	1.78*E* − 131	1.60**E** − 240	0.00**E** + 00
*f*_4_	1.45*E* + 02	4.10*E* + 01	5.72*E* − 18	7.60*E* − 18	1.56*E* − 16	3.00*E* − 16	1.46**E** − 43	3.04**E** − 43
*f*_5_	3.60*E* + 01	1.03*E* + 01	2.42**E** + 01	6.48**E** − 01	2.46*E* + 01	7.50*E* − 01	2.65*E* + 01	1.18*E* + 00
*f*_6_	3.19*E* − 02	1.27*E* − 02	3.55**E** − 15	0.00**E** + 00	3.91*E* − 15	1.12*E* − 15	3.55**E** − 15	0.00**E** + 00
*f*_7_	9.88*E* + 00	2.48*E* + 00	1.31**E** + 01	5.78*E* + 00	1.68*E* + 01	8.12*E* + 00	2.41*E* + 01	1.40**E** + 01
*f*_8_	6.53*E* − 02	1.08*E* − 02	0.00**E** + 00	0.00**E** + 00	0.00**E** + 00	0.00**E** + 00	0.00**E** + 00	0.00**E** + 00
*f*_9_	8.76*E* − 03	3.43*E* − 03	0.00**E** + 00	0.00**E** + 00	0.00**E** + 00	0.00**E** + 00	0.00**E** + 00	0.00**E** + 00

**Table 4:** The mean FEs needed to reach an acceptable solution and reliability “ratio” being the percentage of trial runs reaching acceptable solutions for 10-dimensional functions.

Methods	CLPSO		TLBO		ETLBO		TLBO-PSO	
	mFEs	Ratios	mFEs	Ratios	mFEs	Ratios	mFEs	Ratios
*f*_1_	27041.1	**100.0%**	2744.9	**100.0%**	3044.1	**100.0%**	**1640**	**100.0%**
*f*_2_	NaN	0.0%	5748	**100.0%**	6106.3	**100.0%**	**2858.2**	**100.0%**
*f*_3_	24058.3	**100.0%**	2336.1	**100.0%**	2631.1	**100.0%**	**1449.2**	**100.0%**
*f*_4_	NaN	0.0%	**5999.3**	**100.0%**	6040.2	**100.0%**	**3084.3**	**100.0%**
*f*_5_	NaN	0.0%	45112	20.0%	**43793.5**	**40.0%**	NaN	0.0%
*f*_6_	38089.8	**100.0%**	4201.4	**100.0%**	4577.4	**100.0%**	**2480.4**	**100.0%**
*f*_7_	26783.3	**100.0%**	23632.8	**50.0%**	17727.2	**50.0%**	**12833**	40.0%
*f*_8_	41009.2	**100.0%**	6110.1	**100.0%**	6686.2	**100.0%**	**3278.9**	**100.0%**
*f*_9_	24268	**100.0%**	4123.3	**100.0%**	5467	**100.0%**	**2796.2**	90.0%

**Table 5:** The mean FEs needed to reach an acceptable solution and reliability “ratio” being the percentage of trial runs reaching acceptable solutions for 30-dimensional functions.

Methods	CLPSO		TLBO		ETLBO		TLBO-PSO	
	mFEs	Ratios	mFEs	Ratios	mFEs	Ratios	mFEs	Ratios
*f*_1_	0.0%	3502.8	**100.0%**	4039.5	**100.0%**	**2327.9**	**100.0%**	0.0%
*f*_2_	0.0%	13845.3	**100.0%**	13699.8	**100.0%**	**6802.7**	**100.0%**	0.0%
*f*_3_	0.0%	3214.1	**100.0%**	3716.9	**100.0%**	**2093**	**100.0%**	0.0%
*f*_4_	0.0%	26934.9	**100.0%**	27876.2	**100.0%**	**14067.3**	**100.0%**	0.0%
*f*_5_	0.0%	NaN	0.0%	NaN	0.0%	NaN	0.0%	0.0%
*f*_6_	0.0%	5039.1	**100.0%**	5744.6	**100.0%**	**3305.4**	**100.0%**	0.0%
*f*_7_	0.0%	NaN	0.0%	NaN	0.0%	**4629**	**10.0%**	0.0%
*f*_8_	0.0%	7188.8	**100.0%**	8125.9	**100.0%**	**4315.3**	**100.0%**	0.0%
*f*_9_	**100.0%**	2046.8	**100.0%**	2361	**100.0%**	**1492.5**	**100.0%**	**100.0%**

## References

[1] Floudas C. A., Gounaris C. E. (2009). A review of recent advances in global optimization. *Journal of Global Optimization*.

[2] Kennedy J., Eberhart R. Particle swarm optimization.

[3] Sabat S. L., Ali L., Udgata S. K. (2011). Integrated learning particle swarm optimizer for global optimization. *Applied Soft Computing*.

[4] Shi Y., Eberhart R. A modified particle swarm optimizer.

[5] Pant M., Thangaraj R., Singh V. P. (2009). Particle Swarm optimization with crossover operator and its engineering applications. *IAENG International Journal of Computer Science*.

[6] Ting T.-O., Rao M. V. C., Loo C. K., Ngu S.-S. A new class of operators to accelerate particle swarm optimization.

[7] Li C., Liu Y., Zhao A., Kang L., Wang H. (2007). A fast particle swarm algorithm with cauchy mutation and natural selection strategy. *Advances in Computation and Intelligence*.

[8] Zhang H. (2012). An analysis of multiple particle swarm optimizers with inertia weight for multi-objective optimization. *IAENG International Journal of Computer Science*.

[9] Ali M. M., Kaelo P. (2008). Improved particle swarm algorithms for global optimization. *Applied Mathematics and Computation*.

[10] Zhan Z.-H., Zhang J., Li Y., Chung H. S.-H. (2009). Adaptive particle swarm optimization. *IEEE Transactions on Systems, Man, and Cybernetics, Part B: Cybernetics*.

[11] Zhan Z.-H., Zhang J. (2008). Adaptive particle swarm optimization. *Ant Colony Optimization and Swarm Intelligence*.

[12] Banks A., Vincent J., Anyakoha C. (2007). A review of particle swarm optimization. Part I: background and development. *Natural Computing*.

[13] Banks A., Vincent J., Anyakoha C. (2008). A review of particle swarm optimization. Part II: hybridisation, combinatorial, multicriteria and constrained optimization, and indicative applications. *Natural Computing*.

[14] Rana S., Jasola S., Kumar R. (2011). A review on particle swarm optimization algorithms and their applications to data clustering. *Artificial Intelligence Review*.

[15] Rao R. V., Savsani V. J., Vakharia D. P. (2012). Teaching-learning-based optimization: an optimization method for continuous non-linear large scale problems. *Information Sciences*.

[16] Rao R. V., Savsani V. J., Vakharia D. P. (2011). Teaching-learning-based optimization: a novel method for constrained mechanical design optimization problems. *Computer-Aided Design*.

[17] Rao R. V., Savsani V. J., Balic J. (2012). Teaching–learning-based optimization algorithm for unconstrained and constrained real-parameter optimization problems. *Engineering Optimization*.

[18] Suresh C. S., Anima N. (2011). Data clustering based on teaching-learning-based optimization. *Swarm, Evolutionary, and Memetic Computing: Second International Conference, SEMCCO 2011, Visakhapatnam, Andhra Pradesh, India, December 19–21, 2011, Proceedings, Part II*.

[19] Toĝan V. (2012). Design of planar steel frames using Teaching-Learning Based Optimization. *Engineering Structures*.

[20] Hossein H., Taher N., Seyed I. T. A Modified TLBO algorithm for placement of AVRs considering DGs.

[21] Rao R. V., Patel V. (2012). An elitist teaching-learning-based optimization algorithm for solving complex constrained optimization problems. *International Journal of Industrial Engineering Computations*.

[22] Liang J. J., Qin A. K., Suganthan P. N., Baskar S. (2006). Comprehensive learning particle swarm optimizer for global optimization of multimodal functions. *IEEE Transactions on Evolutionary Computation*.

[23] Zhang W.-J., Xie X.-F. DEPSO: hybrid particle swarm with differential evolution operator.

[24] Meng H.-J., Zheng P., Wu R.-Y., Hao X.-J., Xie Z. A hybrid particle swarm algorithm with embedded chaotic search.

[25] Kaveh A., Talatahari S. (2008). A hybrid particle swarm and ant colony optimization for design of truss structures. *Asian Journal of Civil Engineering*.

[26] Plevris V., Papadrakakis M. (2011). A hybrid particle swarm—gradient algorithm for global structural optimization. *Computer-Aided Civil and Infrastructure Engineering*.

[27] Duan H., Luo Q., Shi Y., Ma G. (2013). Hybrid particle swarm optimization and genetic algorithm for multi-UAV formation reconfiguration. *IEEE Computational Intelligence Magazine*.

[28] Wolpert D. H., Macready W. G. (1997). No free lunch theorems for optimization. *IEEE Transactions on Evolutionary Computation*.

